# Inter-layer synchronization in non-identical multi-layer networks

**DOI:** 10.1038/srep45475

**Published:** 2017-04-04

**Authors:** I. Leyva, R. Sevilla-Escoboza, I. Sendiña-Nadal, R. Gutiérrez, J.M. Buldú, S. Boccaletti

**Affiliations:** 1 Complex Systems Group & GISC, Universidad Rey Juan Carlos, 28933 Móstoles, Madrid, Spain; 2 Center for Biomedical Technology, Universidad Politécnica de Madrid, 28223 Pozuelo de Alarcón, Madrid, Spain; 3 Centro Universitario de los Lagos, Universidad de Guadalajara, Jalisco 47460, Mexico; 4 School of Physics and Astronomy, University of Nottingham, Nottingham, NG7 2RD, UK; 5 CNR-Institute of complex systems, Via Madonna del Piano 10, 50019 Sesto Fiorentino, Italy; 6 The Italian Embassy in Israel, Hamered Street 25, 68125 Tel Aviv, Israel

## Abstract

Inter-layer synchronization is a dynamical process occurring in multi-layer networks composed of identical nodes. This process emerges when all layers are synchronized, while nodes in each layer do not necessarily evolve in unison. So far, the study of such inter-layer synchronization has been restricted to the case in which all layers have an identical connectivity structure. When layers are not identical, the inter-layer synchronous state is no longer a stable solution of the system. Nevertheless, when layers differ in just a few links, an approximate treatment is still feasible, and allows one to gather information on whether and how the system may wander around an inter-layer synchronous configuration. We report the details of an approximate analytical treatment for a two-layer multiplex, which results in the introduction of an extra inertial term accounting for structural differences. Numerical validation of the predictions highlights the usefulness of our approach, especially for small or moderate topological differences in the intra-layer coupling. Moreover, we identify a non-trivial relationship connecting the betweenness centrality of the missing links and the intra-layer coupling strength. Finally, by the use of multiplexed layers of electronic circuits, we study the inter-layer synchronization as a function of the removed links.

Complex networks is one of the most active research topics in today’s nonlinear science^1^. As the field is rapidly evolving (mostly due to the huge amount of data collected nowadays), novel features are incorporated to better describe real world systems. Among these, the extension of the traditional framework to include the *multi-layer* nature of networks has significantly altered the landscape of network science. In a multi-layer description, units can be arranged in several layers (each of them accounting for a different kind of relationship or interaction between the nodes), either simultaneously or in an alternating fashion^2^,^3^,^4^.

On the other hand, synchronization is one of the most relevant dynamical processes encountered in nature, and probably the one that has been most thoroughly studied in the context of complex networks^1^,^5^. Only very recently the study of synchronization has been extended to multi-layers^4^ and, though an exact analytical treatment is available for just particular cases^6^,^7^,^8^,^9^,^10^, several synchronization scenarios have been addressed. Namely, unidirectional coordination between layers^11^,^12^, synchronization control^13^, multi-layer explosive synchronization^14^, synchronization driven by energy transport in interconnected networks^15^, delayed synchronization between layers^16^,^17^ and global synchronization on interconnected layers as in Smart Grids^8^ or neural systems^18^, and networks with multiple interaction layers^19^.

In the majority of these studies, the multi-layer structure of connections supports a global synchronous state in which all the nodes in all the layers behave coherently. More general forms of synchronization, however, are inherently possible on top of a multi-layer structure, as for instance intra-layer synchronization^20^ (where nodes evolve synchronously within each layer but layers do not necessarily evolve coherently), inter-layer synchronization^11^,^21^ (where, instead, layers are synchronized but nodes within each layer are not), and cluster synchronization^22^.

In some of theses studies, diverse formalisms based on the Master Stability Function (MSF) are used for the study of multiplex synchonization in restricted cases^6^,^10^,^13^,^23^. Recently, we have provided analytical, numerical and experimental evidence of *inter-layer* synchronization^21^, based on a version of the MSF for reducing the system dimensionality, and under the assumption that different layers are structurally identical. In this work, we extend the study to the (more realistic) case of nonidentical layers. As main results, we offer a comprehensive (numerical, experimental and analytical) description of the perturbative effects that the deletion of *m* links in one of the layers has on the stability of the inter-layer synchronous state, and show a non-trivial relationship connecting the betweenness centrality of the missing links and the intra-layer coupling strength.

## Results

**Figure 1:**
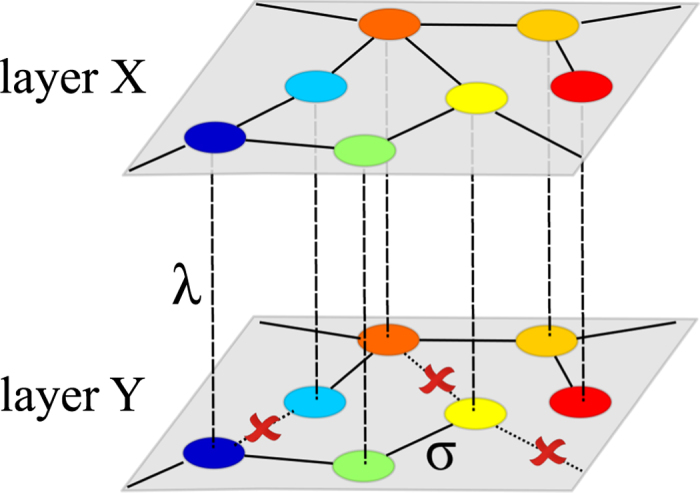
Schematic representation of a multiplex of two layers of identical oscillators. Labels *σ* and *λ* denote the intra- and inter-layer coupling strengths, respectively. Each node *i (j*) in the top (bottom) layer is an *m* dimensional dynamical system whose state is represented by the vector **x**_***i***_ (**y**_***j***_). The topologies of layers *X* and *Y* are encoded in the 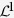 and 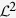 Laplacian matrices, respectively. Originally 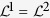, but as we start deleting links, we can write 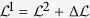 where 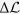 contains the links that have been deleted in the bottom layer.

The object of our study is a multiplex of two layers, formed by *N* identical *m* dimensional dynamical systems, whose states are represented by the *mN* × 1 column vectors 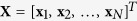 and 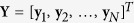 with 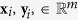 for 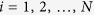. Here, we focus on the case in which the topology of the two layers is different, and encoded by the elements of the Laplacian matrices 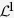 and 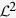 respectively, as depicted in Fig. 1. Therefore, the evolution of the system is given by


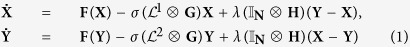


where the function 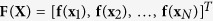, with 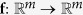 representing the evolution vectorial function. **G**, **H** are the *m *× *m* matrices representing the linear intra (**G**) and inter-layer (**H**) coupling schemes, respectively. The *N* × *N* identity matrix 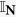 represents the inter-layer topology for the multiplex network. The parameters 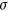 and *λ* are the intra- and the inter-layer coupling strengths.

When the layers are identical (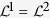), the inter-layer synchronous evolution (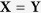) is a solution of Eq. (1), independently of the existence of intra-layer synchronization^21^. When the inner structure of the layers differs (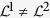), however, 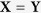 is no longer a solution of Eq. (1) –i.e. the system may satisfy that condition at a given time if e.g. the two layers start from the same initial condition, but the dynamics will move away from the synchronization manifold 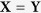, which is no longer an invariant set of the dynamics. Yet, it can be expected that when the topologies of the two layers are actually *similar* (i.e. when their difference is limited to only a few links), one can proceed with an approximation, which consists in supposing that the dynamics of the system would anyway visit regions of the state space sufficiently close to 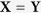, so that the predictive use of the Master Stability Function (MSF) methodology^1^,^24^ still makes sense. In the Methods section, the interested reader can find the details of such an approximate MSF approach, whose predictions are tested in the following, both numerically and experimentally. It is, in any case, important to remark that our approach relies on approximations that are not fully controllable, and therefore it is reasonable to expect that predictions based on the associated conditional Lyapunov exponents would less and less quantitatively fit the real evolution of the system, the more the two layers differ in the structure of connectivity. The validity of the approximation is checked by monitoring the value of the inter-layer synchronization error, which is defined as 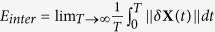, where 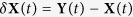 is the vector describing the difference between the layers’ dynamics and 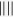 stands for the Euclidean norm. Additionally, the intra-layer errors 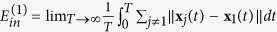 and 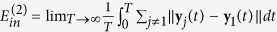, and their difference 
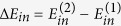
 will be computed when they may help to elucidate the dynamics under investigation.

### Numerical results

The first goal is to numerically assess the range of validity of the approximate MSF approach detailed in the Methods section. For this purpose, the two layers are initially created as identical, and then structural differences are generated by removing *m* links in 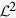. To evaluate the range of impact of the structural differences, we have chosen the *m* links to be removed following an *edge betweenness* criterion^25^, but other criteria will be discussed later. Accordingly, each simulation is repeated twice, a first time removing the links with the highest edge betweenness (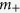), and a second time removing those that have the lowest edge betweenness (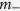). The procedure never produces a lack of connectedness in the graphs (for the networks and number of removals considered), and in case of degeneracy, a link is chosen at random among those that have the same betweenness. Without lack of generality, we consider two possible kinds of topologies where both layers are either Erdös-Rényi^26^ (ER) or scale-free^27^ (SF), in both cases with *N* = 500, and average degree 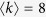.

Nodes are here Rössler oscillators^28^, whose autonomous evolution is given by 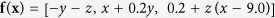. ER and SF networks are generated by means of the procedures proposed in refs 26 and 27, respectively, and therefore the considered SF networks display a degree distribution 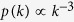.

In our first example, the intra- and inter- layer local coupling functions are set to be 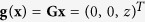 and 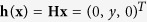 respectively, so that (according to the standard MSF classification established in ref. 1) the intra-layer configuration is within class I (and, therefore, intra-layer synchronization is never possible), whereas the inter-layer configuration corresponds to class II (i.e., synchronization may be stable when the coupling strength exceeds a certain threshold).

**Figure 2:**
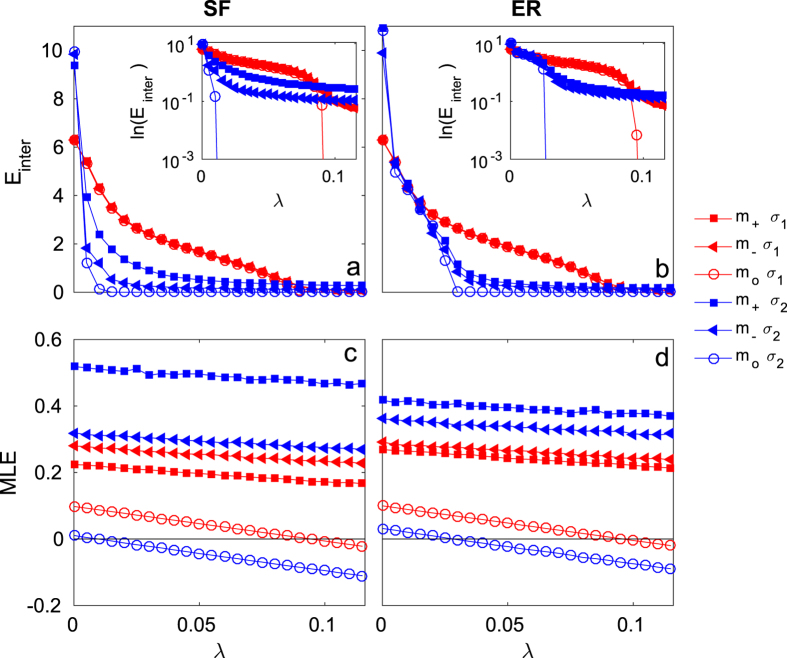
Results for inter-layer dynamics as a function of the intra-layer coupling strength *λ* for class-I layers. *E*_*inter*_ (see main text) in multiplexes of SF (**a**) and ER (**b**) layers of *N* = 500 Rössler oscillators, for two different values of intra-layer coupling *σ*_1_ = 0.1 (red symbols) and *σ*_2_ = 1.0 (blue symbols) when the 50 links with larger (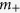, 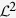) and lower (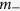, ▲) betweenness are removed from 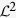, and for identical layers (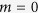, ○). Insets: Detail of the respective panels (**a**) and (**b**), in semi-logarithmic scale. (**c**,**d**) The corresponding MLE for the approximate expression in Eq. (9).

In Fig. 2 we show the *E*_*inter*_ (panels a and b) and MLE (panels c and d) as a function of the inter-layer coupling *λ* for two different values of intra-layer coupling *σ*_1_ = 0.1 (red curves) and *σ*_2_ = 1.0 (blue curves) when the 50 links (i.e. approximately 2.5% of the total number) with the largest (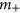, squares) and lowest (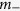, triangles) betweenness centrality values are removed from the SF (Fig. 2a,c) and ER (Fig. 2b,d) 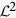 layers. For the sake of comparison, we also report the curves for the case of identical layers (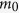, circles).

It can be observed that, in spite of the nonidentical layer topologies that make complete synchronization formally impossible, the *E*_*inter*_ series presents, in fact, apparently small differences with the identical case for both 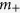 and 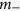 and for the chosen *σ* values, which can be better appreciated in a logarithmic representation (as shown in the insets of the corresponding figures). Independently of the layer topology, at relatively large *σ (σ*_2_), *E*_*inter*_ is seen to follow more closely the trend observed in the identical case when compared with smaller values of *σ (σ*_1_). This form of resilience is in agreement with the fact that the non identity of the layers results in the presence of an inertial term, which depends indeed on the value of *σ* (see details in the Methods section). The corresponding Maximum Lyapunov Exponent (MLE) is shown in the bottom panels of Fig. 2, confirming the behavior of the inter-layer dynamics depicted in the upper panels. Notice that the effects of removing links with high or low betweenness are more pronounced in multiplexes made of SF layers than in those made of ER ones. Another observation, which will be further highlighted in the following, is that the impact on the inter-layer synchronization of removing high or low betweenness links is reversed depending on the strength of the intra-layer coupling: in both the ER and SF cases, removing 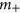 links deteriorates (improves) the synchronization levels with respect to removing 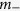 links for large (small) *σ* values.

**Figure 3:**
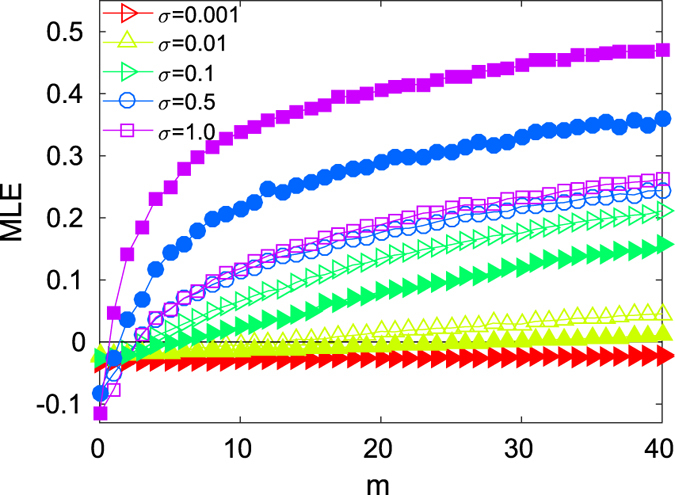
Maximum Lyapunov exponents (MLE) for different intra-coupling strengths *σ* as a function of the number of links removed *m*, for the cases in which the removed links have the highest (full markers) or the lowest (void markers) edge betweenness. Layers are SF and of class I with *N* = 500 Rössler oscillators and *λ* = 0.12.

An analysis that further elucidates the role of the structural differences is provided in Fig. 3, where the dependence of MLE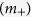 and MLE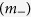 is reported as a function of *m*, for a fixed value of *λ* (at which there is inter-layer synchronization for *m* = 0). As predicted by the approximated MSF approach, the dynamics drifts from the identical case at smaller values of *m*, as *σ* increases. We here find a unexpected and interesting feature, already glimpsed before in Fig. 2, that entangles the intra-layer structure with the inter-layer dynamics: for small values of *σ*, removing the 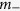 lowest betweenness centrality links results in a stronger perturbation for the inter-layer synchronization than removing the same number 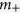 of highest betweenness links. However, for larger values of *σ*, the effect is reversed.

**Figure 4:**
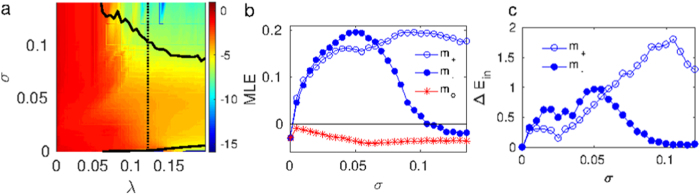
(**a**) Contour map of log(*E*_*inter*_) in the (*σ, λ*) parameter space with 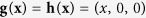 (class III in the intra- and inter-layer dynamics) and *m*_−_ = 50 links removed from 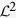. The black contour line corresponds to the isoline where the MLE changes its sign from positive to negative. Color code is shown in the lateral bar. (**b**) MLE vs. *σ* for fixed *λ* = 0.12 (corresponding to the dotted line in the left panel) where the 50 links with largest (*m*_+_ = 50, 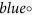) and lowest (*m*_−_ = 50, ●) betweenness are removed from 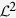. The identical case 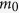 (*) is also plotted for comparison. (**c**) Averaged intra-layer error difference 
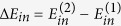
 for the same parameter values as in (**b**). In all panels, the two layers are ER of *N* = 500 Rössler oscillators and 〈*k*〉 = 8. Each point is an average over 5 realizations.

We tested also the case 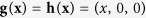 (where the MSFs belong to class III for both the intra- and inter-layer dynamics). The validity of our approximation is shown in the left panel of Fig. 4, where log(*E*_*inter*_) is plotted for the 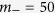 case in the (*σ*, λ) parameter space. The limit in which the MLE becomes negative (black solid line) closely corresponds to 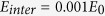, *E*_0_ being the corresponding inter-layer synchronization error for *λ* = 0 (uncoupled layers) at each *σ* value. Once again, our approximated MSF provides an excellent reference for the analysis of the nonidentical inter-layer dynamics. In particular, in Fig. 4 we compare the MLE curves as a function of *σ* in three different scenarios: identical layers (stars) and nonidentical layers after removing 50 links with the lowest (full circles) or the highest (empty circles) betweenness. In all cases, *λ* was fixed to 0.12 (which makes the two layers synchronizable when they are identical). For weakly coupled layers (low values of *σ*), the perturbation caused by removing 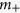 or 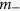 links is similar, but as the intra-layer coupling increases, the multiplex is able to recover the inter-layer synchronization state despite the 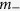 links that have been removed from one of the layers, while it is never again achieved in the case of removing the largest betweenness links.

In order to understand the remarkable influence of choosing to remove the 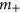 links or the 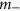 links on the emergence of inter-layer synchronization, we checked the impact of this choice on the *intra-layer* dynamics. Removing the links with the largest betweenness affects the information flow in the network and therefore, for the same value of *σ*, the layer coherence will deteriorate more by this action than by removing the links with the lowest betweenness. In Fig. 4c we plot the corresponding intra-layer errors difference 
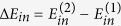
 of Fig. 4b. As it can be observed, the two curves follow perfectly the same trends as the inter-layer MLEs shown in Fig. 4b, disclosing the close relationship between the inter and the intra-layer dynamics.

**Figure 5:**
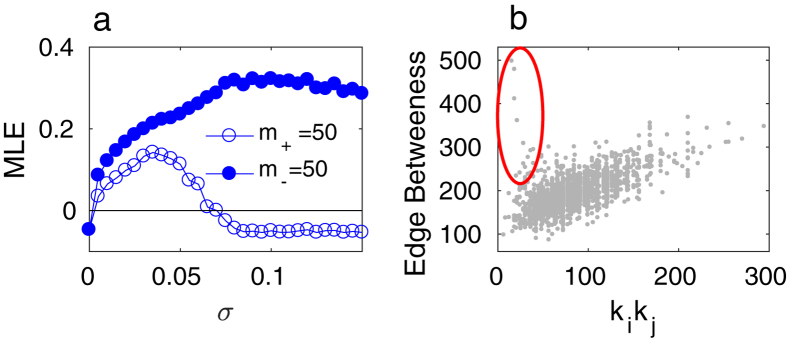
(**a**) MLE vs. *σ* for the same parameter values as in Fig. 4, where the 50 links with the largest (*m*_+_ = 50, ○) and lowest (*m*_−_ = 50, ●) degree product *k*_*i*_, *k*_*j*_ are removed from 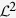. (**b**) Correlation between the degrees’ product *k*_*i*_, *k*_*j*_ and edge betweenness for all the links in a sample network used in (**a**). The red ellipse marks those nodes which simultaneously have high edge betweenness and low *k*_*i*_, *k*_*j*_.

So far, we have chosen the edge betweenness criterion to provide an insight on how the absence of certain links would impact the inter-layer synchronization. However, the edge betweenness is not the only possible criterion to rank the importance of the links in governing the multiplex dynamics. In Fig. 5a we report the results corresponding to the same settings used in Fig. 4b but applying this time the criterion of the product of the nodes’ degree, 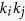, to remove the edge connecting nodes *i* and *j*. The effect on the inter-layer coherence of removing links following this criterion resembles that resulting from applying the edge betweenness criterion but with a substantial difference: while in the latter case, the links whose perturbative effect after removal become more perturbative by increasing the intra-layer coupling *σ* are those with the highest betweenness, in the former case the inter-layer synchronization become more affected when removing the links connecting the nodes whose degrees product is the lowest. This reverse effect is explained in Fig. 5b where we compare 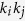 vs edge betweenness for all the links of a typical ER network used in our study and the links with the highest betweenness happen to be also those with a low 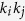 (red ellipse). Therefore, the results based on the two criteria are fully consistent.

### Experimental results

**Figure 6:**
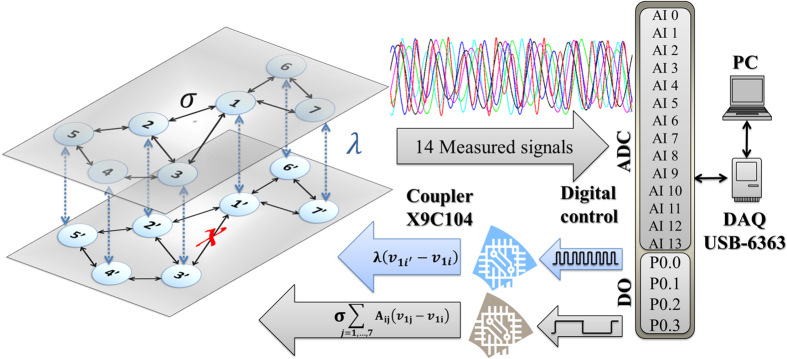
Experimental setup. The left image is a sketch of the coupling topology of the 14 electronic circuits composing the multiplex network (see main text for the description of the experimental procedure used). The whole experiment is controlled from a PC with Labview Software.

Our predictions can be substantiated by evidence resulting from an experiment with electronic circuits. The setup, sketched in Fig. 6, consists of an electronic array, a personal computer (PC), 14 analog to digital converters (ADC) and 4 digital ports (DO) from a multi-functional data card (DAQ) controlled by Labview. The ADCs are used for sampling one of the state variables out of all the networked circuits, while the DOs are used as controllers for the gain of the two coupling strengths *σ* and *λ*. The array is made of 14 Rössler-like circuits arranged in two layers (blue nodes), each one of them having two different electronic couplers, one for the coupling among nodes in the same layer (*σ*) and the second for the interaction of each node with its replica in the other layer (*λ*). The layers are identical but for a single lacking link in one of the networks, which can be chosen to be any link in the experiment.

The chaotic dynamics of the Rössler-like circuits is well approximated by an electronic model, where the nodes’ state variables *x, y, z* are translated into three different voltages ν_1_, ν_2_ and ν_3_ that evolve in time as follows:


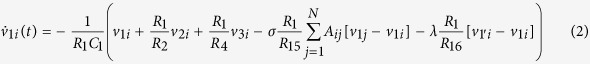



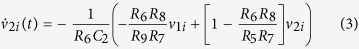



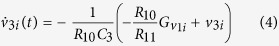


where 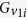 is a nonlinear gain function given by:


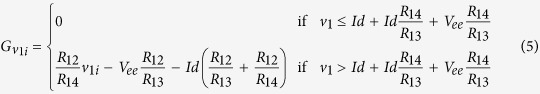


and *C*_*i*_ and *R*_*i*_ are a series of capacitors and resistances whose values are summarized in Table 1 (the interested reader can check refs 29 and 30 for a detailed description of the experimental implementation of the Rössler-like system, and at refs 9, 21 and 31 for previous realizations with different network configurations).

**Figure 7:**
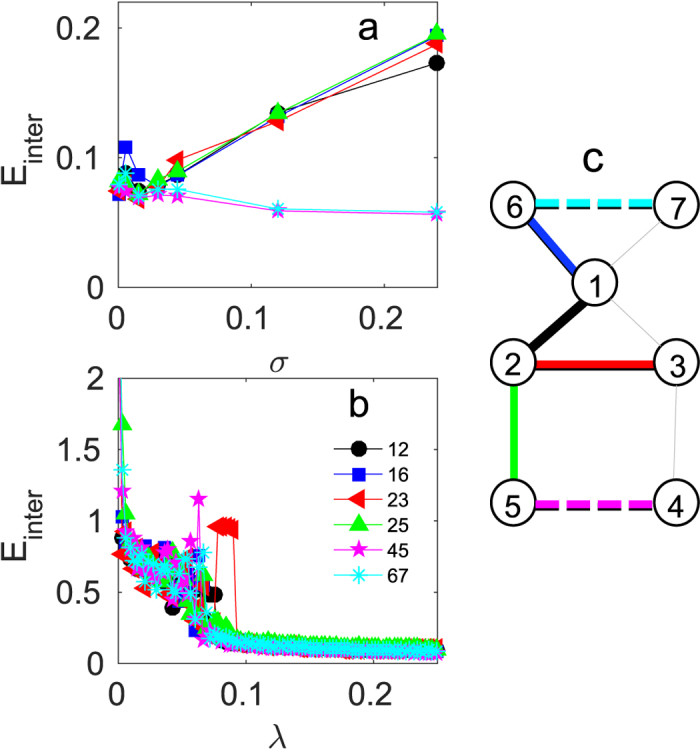
Experimental results for a perturbed multiplex network of electronic Rössler oscillator. (**a**) *E*_*inter*_ as a function of *σ* for *λ* = 0.3 and (**b**) *E*_*inter*_ as a function of *λ* for *σ* = 0.05 for each case when one of the links (see color code in the legend of panel (**b**)) in one of the layers is removed. The color of the symbols corresponds to the color of the links in the layer structure scheme in panel (**c**). High (low) edge betweenness links are highlighted with continuous (dashed) lines.

Departing from the initial network configuration of Fig. 7c, we carry out a series of experiments where, a link (each time a different one) is removed from one of the layers (always the same one). The removed link between nodes *i* and *j* will be referred to in the following as (*ij*). Both *σ* and *λ* values are initially set to zero, and the polarization voltage of the circuits is turned off and on, after a waiting time of 500 ms. The signals corresponding to the *x* state variables of the 14 circuits are acquired by the analogue ports AI0–AI13 and saved in the PC for further analysis. For every *σ* value, *λ* is then incremented by one step, and the procedure is repeated 100 times (until the maximum value of *λ* is reached). When the entire run is finished, *σ* is increased by one step, and another cycle of *λ* values is initiated. The whole procedure is repeated for every link of the network.

The experimental results for *E*_*inter*_ are presented in Fig. 7a,b, which confirm our predictions on the impact on the inter-layer dynamics of the removal of links with high or low betweenness. In Fig. 7a, as *σ* is increased while keeping constant *λ* = 0.3, the effect of deleting the links with the highest betweenness [links (12),(16),(23) and (25) in our example, see network scheme in panel c for reference] leads to a conspicuous increase in *E*_*inter*_. A very different behavior is observed when we remove the links with lower betweenness [(45) and (67)], which consistently decreases the inter-layer error as the intra-layer coupling strength increases. This is in full qualitative agreement with what observed in the numerical counterpart (see Fig. 4), and confirms the entanglement between the intra-layer structure and the inter-layer dynamics. Figure 7b reports the dependence of *E*_*inter*_ on *λ* when *σ* = 0.05, showing that the network can reach a quasi-synchronous state even in the presence of structural defects, as predicted in Fig. 4. Also note the robustness of the theoretical predictions despite the intrinsic parameter mismatch (~5%) of the electronic components.

## Discussion

These results allow us to draw a series of important conclusions about the effects of structural layer differences on the capability of multiplex networks to display synchronized layers, with nodes in each layer which do not necessarily evolve in unison. It is important to remark that the study of inter-layer synchronization was restricted so far to the case in which all layers had an identical connectivity structure. When layers are not identical, several conceptual issues arise, the most relevant being that the inter-layer synchronous manifold is no longer invariant under the dynamics, and one has therefore to proceed with approximate treatments.

We have demonstrated that an approximate analytical treatment of a two-layer multiplex results in the introduction of an extra inertial term accounting for structural differences. The predictions have been validated numerically and, most importantly, by means of an experiment with electronic circuits. The conclusion is that, even in this case in which layers are not identical and the exact synchronized solution does not exist, the approximate Master Stability Function is a very good tool to study the inter-layer dynamics of multiplex networks. Using such a framework, indeed, we could predict the effect that missing links in one of the layers has on the inter-layer synchronization, evidencing a non-trivial relationship between the edge centrality of the different links and the balance between intra- and inter-layer couplings.

The fact that the predictions are solidly verified in an experimental setup (where fluctuations, noise and uncertainty of nodes’ parameters are unavoidable) highlights the robustness of our analytical predictions. Our results can provide a starting point for the study of inter-layer synchronization in even more general configurations, as unidirected networks^13^ or general multilayer networks.

## Methods

### Approximate Master Stability Function (MSF) formalism for a two-layer network

We here summarize the main steps of the perturbation analysis of Eq. (1). First, one can always define 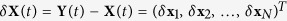 and calculate its law of motion


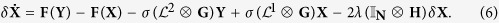


Notice that the existence of a perfect synchronous inter-layer solution means that 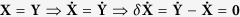. Introducing these equivalences into Eq. (6), it leads:


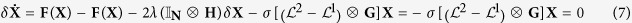


which in principle is true if and only if *σ* = 0 (isolated nodes) or 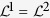, that is, the layers are identical. In other words, we can conclude that the solution 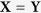 for all times is not compatible with Eq. (1).

Second, one can define 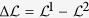, as the matrix representing the difference between the two Laplacians. Plugging 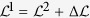 into Eq. (6), one obtains the following dynamics at the level of individual nodes:


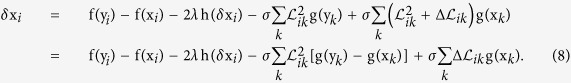


However, let now assume that, in a large enough network the effect of the perturbation 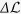 is small enough for an inter-layer almost synchronous dynamics 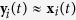 to emerge. Then, one can take 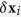 to be small quantities, and expand to first order around 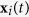. The equations resulting from the linearization are:


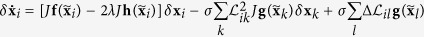


where 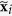 is the state of node *i* in an isolated layer evolving according to 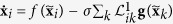.

By comparing this result with the identical case^21^, it can be seen that the non-identity of the systems is reflected in the last *inertial* term, whose role in pushing the dynamics away from the identical case is expected to become more prominent when the topological differences are large. Additionally, it predicts that the divergence from the inter-layer synchronization will depend on the the intra-layer coupling strength, which is in its own right an interesting result on the rich interplay between intra-layer and inter-layer effects, an aspect of inter-layer synchronization that was thoroughly explored in the identical case in ref. 21. Following the MSF approach, a negative sign in the maximum conditional Lyapunov exponent (MLE) obtained from Eq. (9) can be taken as an indication for the presence of inter-layer synchronization^21^.

## Additional Information

**How to cite this article:** Leyva, I. *et al*. Inter-layer synchronization in non-identical multi-layer networks. *Sci. Rep.* **7**, 45475; doi: 10.1038/srep45475 (2017).

**Publisher's note:** Springer Nature remains neutral with regard to jurisdictional claims in published maps and institutional affiliations.

## Figures and Tables

**Table 1:** Values of the electronic components used for the construction of the electronic version of the Rössler-like system.

*C*_1_ = 4.7 nF	*C*_2_ = 4.7 nF	*C*_3_ = 4.7 nF	*σ* = [0 − 0.25]
*R*1=2 *M*Ω	*R*2 = 200 *K*Ω	*R*3 = 10 *K*Ω	*R*4 = 100 *K*Ω
*R*5 = 50 *K*Ω	*R*6 = 5 *MK*Ω	*R*7 = 100 *K*Ω	*R*8 = 10 *K*Ω
*R*9 = 10 *K*Ω	*R*10 = 100 *K*Ω	*R*11 = 100 *K*Ω	*R*12 = 150 *K*Ω
*R*13 = 68 *K*Ω	*R*14 = 10 *K*Ω	*R*15 = 100 *K*Ω	*R*16 = 100 *K*Ω
*RC* = *R*3 + *R*5	*Id* = 0.7	*V*_*ee*_ = 15	*λ* = [0 − 0.25]
